# A Web-Based Self-Help Psychosocial Intervention for Adolescents Distressed by Appearance-Affecting Conditions and Injuries (Young Persons’ Face IT): Feasibility Study for a Parallel Randomized Controlled Trial

**DOI:** 10.2196/14776

**Published:** 2019-11-22

**Authors:** Heidi Williamson, Claire Hamlet, Paul White, Elsa M R Marques, Thomas Paling, Julia Cadogan, Rohan Perera, Nichola Rumsey, Leighton Hayward, Diana Harcourt

**Affiliations:** 1 Faculty of Health and Applied Sciences University of the West of England Bristol United Kingdom; 2 Faculty of Environment and Technology University of the West of England Bristol United Kingdom; 3 Bristol Medical School University of Bristol Bristol United Kingdom; 4 University Hospitals Bristol NHS Foundation Trust Bristol United Kingdom; 5 Bristol Clinical Commissioning Group Bristol United Kingdom

**Keywords:** physical appearance, body image, disfigurement, visible difference, adolescents, young people, psychological support, online intervention.

## Abstract

**Background:**

Disfigurement (visible difference) from wide-ranging congenital or acquired conditions, injuries, or treatments can negatively impact adolescents’ psychological well-being, education and health behaviours. Alongside medical interventions, appearance-specific cognitive behavioural and social skills training to manage stigma and appearance anxiety may improve psychosocial outcomes. YP Face IT (YPF), is a Web-based seven session self-help program plus booster quiz, utilising cognitive behavioural and social skills training for young people (YP) struggling with a visible difference. Co-designed by adolescents and psychologists, it includes interactive multimedia and automated reminders to complete sessions/homework. Adolescents access YPF via a health professional who determines its suitability and remotely monitors clients’ usage.

**Objective:**

To establish the feasibility of evaluating YPF for 12-17 year olds self-reporting appearance-related distress and/or bullying associated with a visible difference.

**Methods:**

Randomized controlled trial with nested qualitative and economic study evaluating YPF compared with usual care (UC). Feasibility outcomes included: viability of recruiting via general practitioner (GP) practices (face to face and via patient databases) and charity advertisements; intervention acceptability and adherence; feasibility of study and data collection methods; and health professionals’ ability to monitor users’ online data for safeguarding issues. Primary psychosocial self-reported outcomes collected online at baseline, 13, 26, and 52 weeks were as follows: appearance satisfaction (Appearance Subscale from Mendleson et al’s (2001) Body Esteem Scale); social anxiety (La Greca’s (1999) Social Anxiety Scale for Adolescents). Secondary outcomes were; self-esteem; romantic concerns; perceived stigmatization; social skills and healthcare usage. Participants were randomised using remote Web-based allocation.

**Results:**

Thirteen charities advertised the study yielding 11 recruits, 13 primary care practices sent 687 invitations to patients on their databases with a known visible difference yielding 17 recruits (2.5% response rate), 4 recruits came from GP consultations. Recruitment was challenging, therefore four additional practices mass-mailed 3,306 generic invitations to all 12-17 year old patients yielding a further 15 participants (0.5% response rate). Forty-seven YP with a range of socioeconomic backgrounds and conditions were randomised (26% male, 91% white, mean age 14 years (SD 1.7)); 23 to YPF, 24 to UC). At 52 weeks, 16 (70%) in the intervention and 20 (83%) in UC groups completed assessments. There were no intervention-related adverse events; most found YPF acceptable with three withdrawing because they judged it was for higher-level concerns; 12 (52%) completed seven sessions. The study design was acceptable and feasible, with multiple recruitment strategies. Preliminary findings indicate no changes from baseline in outcome measures among the UC group and positive changes in appearance satisfaction and fear of negative evaluation among the YPF group when factoring in baseline scores and intervention adherence.

**Conclusions:**

YPF is novel, safe and potentially helpful. Its full psychosocial benefits should be evaluated in a large-scale RCT, which would be feasible with wide-ranging recruitment strategies.

**Trial Registration:**

ISRCTN registry ISRCTN40650639; http://www.isrctn.com/ISRCTN40650639

## Introduction

### Background

Approximately 1 in 44 individuals has a condition or injury that noticeably affects the appearance of their face, skin, or body shape [1]. Referred to as visible differences, these distinct changes result from congenital (eg, cleft lip and birthmark), neurological (eg, facial palsy), genetic (eg, neurofibromatosis), or acquired conditions (eg, acne). Advances in life-saving treatments are also increasing survivorship associated with an altered appearance resulting from traumatic injury (eg, burn) and disease (eg, meningitis). Appearing *different* in a society that venerates looks can have profound effects during adolescence, a vulnerable period when social comparison with peers/celebrities is high, romantic interest is burgeoning, and appearance impacts self-esteem [2]. Research shows commonalities in the experiences of young people (YP) with a variety of appearance-altering conditions [3]; 30% to 50% struggle with social stigma (eg, teasing, bullying, peer rejection, and unwanted attention from strangers [4]) and/or experience appearance-related distress [5]. If not addressed, these experiences can lead to low self-esteem, social anxiety and avoidance [6,7], poor social and emotional development [8], reduced school performance [9], difficulties with romantic relationships [10], unemployment [11], depression [12], and self-harm and suicidality [13], a health, social, and economic burden to society.

Although surgical and medical advances to ameliorate appearance-altering conditions are advancing, they are not a cure-all [3], and contrary to expectations, the severity, cause, and location of a visible difference do not reliably predict distress [14]. Adjustment is largely determined by intervening sociocognitive factors, including perceived satisfaction with social support and acceptance, Fear of Negative Evaluation (FNE) by others, and social confidence [15]. These factors are potentially amenable to change via psychosocial interventions that offer an adjunct or alternative to medical/surgical solutions and provide skills to tackle stigmatization and appearance-related distress.

Research [16] points to a dearth of evidence-based, cost-effective, and appearance-specific interventions for YP. Within UK primary health care, these YP rarely meet criteria for referral to Child and Adolescent Mental Health Services or waiting lists are long, and those receiving secondary health care for their condition often have no/limited access to psychological support [17]. Stakeholders (eg, clinicians and parents) also report barriers preventing YP from seeking or accepting psychological, particularly face-to-face, support around such a sensitive issue. These include traveling to specialist appointments, fear of further stigmatization, and social anxiety/avoidance [18]. Acknowledging that number of YP experiencing poor mental health is increasing as psychological services are rationed, the United Kingdom’s National Health Service (NHS) has called for innovative and cost-effective interventions that promote self-management and resilience [19]. An appearance-specific Web-based psychosocial intervention could broaden access to support for those with appearance-related distress and improve quality through evidence-based standardized care.

In adults with a visible difference, a randomized controlled trial (RCT) of a multisession Web-based intervention (Face IT) has proved beneficial. Centered on Kent’s Integrated Model of Psychosocial Distress and Intervention for Individuals with Visible Differences [20], Face IT integrated cognitive behavioral therapy (CBT) and social skills training (SST), reduced anxiety-related concerns, and was comparable with face-to-face CBT [21]. Following the Medical Research Council framework for the development of complex interventions [22], the authors worked with YP to co-design an age-appropriate and guided self-help Web-based intervention (Young Person’s Face IT or YP Face IT) based on Face IT [18]. YP Face IT (YPF) is for 12- to 17-year-olds with any appearance-affecting condition who are experiencing social stigma and/or appearance-related distress.

This paper reports the results of a study, which explored the feasibility of evaluating YPF compared with usual care (UC) using an RCT design and provided data to estimate the parameters required to design a definitive trial. There is no standardized treatment for this patient group, and the type and frequency of UC were therefore recorded. The feasibility of recruiting participants via primary care and charitable organizations was also examined. General practitioners (GPs) are accessible to most YP and parents, and charities for those with a wide range of appearance-altering conditions are approached by parents or YP for advice [18]. Both could provide immediate access to evidence-based appearance-related support, including while the YP is waiting for, or to preclude, referral to secondary care services.

### Objectives

The objectives of this study were as follows: (1) to estimate the numbers of eligible participants recruited via primary care practices and charities, including reasons for nonparticipation; (2) to assess participants’ views on study design; (3) to determine the acceptability of the YPF intervention and adherence as well as safeguarding processes; (4) to determine the completion of outcome and resource use measures (for future economic evaluation); (5) to determine the variation of UC provided; (6) to assess the responses to patient-reported outcome measures, to inform the selection of a primary outcome measure and test for harm and potential effectiveness of YPF (the trial was not powered to test statistically significant impact); and to estimate the sample size for a definitive trial.

## Methods

### Trial Design

This parallel-group, randomized controlled feasibility trial compared YPF plus UC with UC only (control) and included a nested economic and qualitative study and online pre- and postassessments at 13, 26, and 52 weeks after randomization. Data analysts (PW, EM, and TP) were blind to group allocation, whereas participants were not. The trial was preregistered, and full protocol published [23]. Ethics approval was given by the UK National Research Ethics Service Committee South West (Ref 14/SW/0058).

### Recruitment

Recruitment was via GP practices and charitable organizations supporting those with a range of appearance-altering conditions (eg, the UK’s Cleft Lip and Palate Association; www.clapa.com). Charities promoted the study via their websites or newsletters. Advertisements were designed alongside service users’ involvement, outlined the study, and included the research team’s contact details.

GP practices were briefed on the study protocol in a 30-min session. Practices used a medical diagnosis coding system to identify eligible patients with an appearance-affecting condition and excluded those deemed unsuitable (eg, condition resolved). Identified YPs were posted a personal invitation and information sheet. For those aged younger than 16 years, letters were addressed to parents/carers who were asked to discuss participation with their child. A reminder, sent 4 weeks later to nonrespondents, included a response form to indicate why they declined and a study-addressed envelope. Staff were also encouraged to introduce the study to potential participants during consultations and provide a leaflet.

In a user-involvement meeting, GPs noted that database records were inaccurate, and they had difficulties identifying eligible patients. Therefore, in a change to the published protocol, subsequent GP practices that joined the study used mass mail out to all their patients aged 12 to 17 years using an online mail management solution (www.cfhdocmail.com); rather than GPs deciding who to invite, all 12- to 17-year old patients could decide on their eligibility. Letters were addressed to parents/carers of those aged younger than under 16 years, as above.

Interested YP/parents contacted the research team who answered questions and confirmed eligibility with the YP (including parent/carer if YP aged <16 years) via the telephone. Informed consent was obtained by participants completing and posting a consent form or verbally consenting via a recorded telephone call.

### Participants

When developing YPF, we sought advice from YP, parents, and health professionals regarding the age range of the intervention’s target audience and other eligibility criteria [18]. Eligible YP were 12- to 17-year-old UK residents with any appearance-affecting condition who self-identified as experiencing appearance-related distress, teasing or bullying, and were fluent in English (YPF has a reading age of 12 years, and audio clips are available on YPF for those who struggle reading text), with internet literacy and access to an internet-enabled device. YP were ineligible if they had a registered learning disability, a diagnosis of clinical depression, psychosis, eating disorder, and posttraumatic stress disorder (PTSD) or were within 12 months of a traumatic injury. PTSD is a risk for those disfigured through trauma [24]. Those aged younger than 16 years required a parent/carer to join the study, and those aged 16 and 17 years were encouraged to inform and involve their parent/carer, but this was not mandatory. Practice staff provided views on recruitment procedures and supervising their patients using YPF.

### Intervention

**Figure 1:**
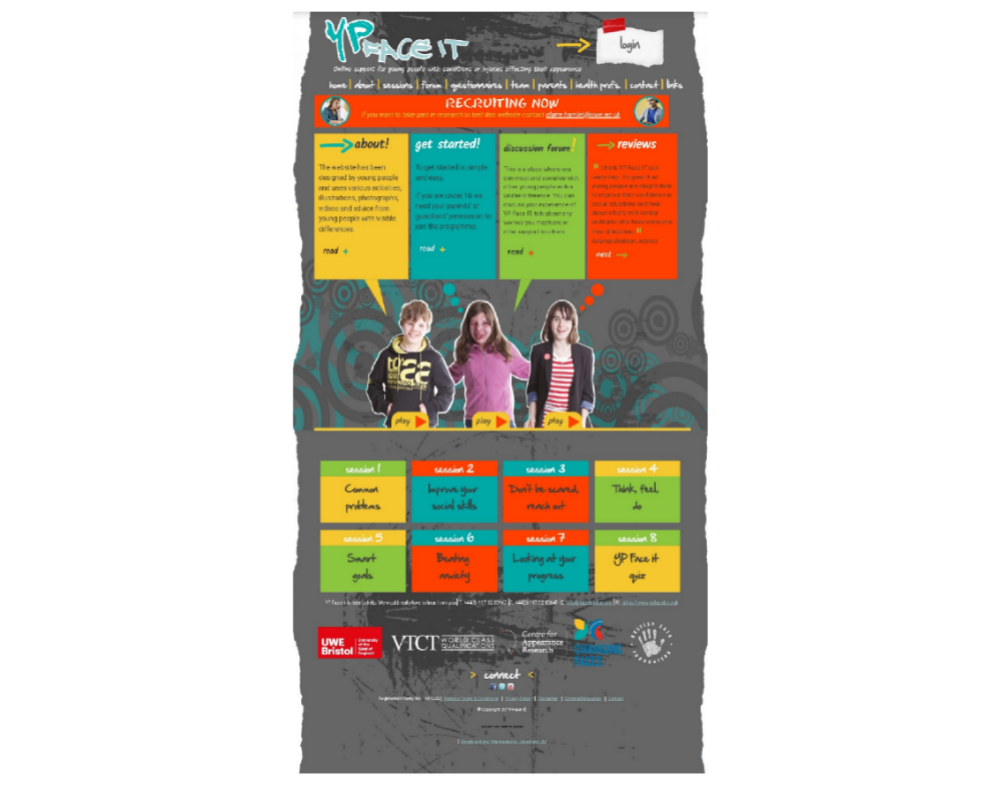
Young Persons’ Face IT homepage.

YPF was developed by the Centre for Appearance Research, is owned by the University of the West of England, and is hosted by Dataphiles plc (www.dataphiles.co.uk). Details of creators and affiliations were provided on the homepage. The participatory action approach used to develop YPF was reported elsewhere [18]. Version 3 (www.ypfaceit.co.uk) was used in this trial during which the content was frozen, and program glitches addressed. The YPF homepage (Figure 1) is freely accessible to all (only the sessions require a personal login) and provides easy-to-understand videos describing the intervention for YP and comprehensive details of the therapeutic content for health professionals.

YPF aims to help YP overcome social anxiety, manage social stigma, and reduce negative thoughts about their appearance that can lead to unhelpful behaviors. It has 7 weekly sessions (each taking approximately 30-40 min to complete) including homework (eg, to practice strategies for managing teasing), and a booster session (quiz) completed 6 weeks later. Sessions are summarized in Table 1 with more detail in the YPF development and protocol papers [18,23]. YPF has a restricted administration area where user accounts are set up by a supervising health professional, and usage is recorded (eg, date and duration of access, pages viewed, and text/numeric responses to embedded reflective and homework activities/quizzes). YP can use a journal that stores personal data and quiz/survey responses and a closed forum to share and receive advice from fellow participants, moderated Monday to Friday by researchers. Participants were allocated a participant identification number, and data were protected via a secure portal using 128-bit Secure Sockets Layer encryption. Users are provided with an e-mail address to report glitches. To check for safeguarding issues (eg, disclosure of abuse, suicidality, and intervention-related adverse events), researchers with safeguarding training (eg, www.nsahealth.org.uk) reviewed users’ activity weekly. The feasibility of nominated staff at 6 GP practices performing this task for their patients was assessed; they received 10-min training and a prompt sheet detailing how to access the administration area and were advised to follow their safeguarding protocols and note actions on the website. Researchers also recorded and referred concerns to the team’s clinical psychologist who decided what, if any, additional support was required.

**Table 1:** Content of Young Persons’ Face IT.

Session title	Session description
Common problems	Common difficulties and feelings experienced by young people with visible differences, shared experiences from similar others, and a review of helpful and unhelpful coping strategies.
Improve your social skills	Using positive body language and talking skills to promote self-confidence and manage negative reactions from others.
Don’t be SCARED, REACH OUT	Recognizing the impact of one’s behavior on others and using the *REACHOUT* toolbox to manage social stigma and challenging situations (Reassurance, Effort and Enthusiasm, Assertiveness, Courage, Humor, Over there, Understanding, and Try again). Interactive videos allow users to practice new techniques.
Think, Feel, Do	Introducing the link between thoughts, feelings, and actions; the common misconceptions young people with visible differences have about the thoughts and actions of others; tips on how to challenge negative thoughts using *catch it; check it; change it*. Users practice this process using interactive social scenarios.
SMART goals	Realistic and achievable goal-setting to overcome social anxiety and to combat self-imposed limitations. Goal-setting examples and testimonials from positive role models. Option to explore issues around romantic relationships.
Beating anxiety	Symptoms of anxiety; anxiety management techniques; using *testing the water* and the *fear ladder* techniques to overcome social anxiety and achieve goals, creating their own fear ladder and setting goals.
Looking at your progress	Revision session on whole program.
Booster quiz	Interactive quiz on key learning points. Facility to identify and revisit areas that the user is struggling with or wishes to revise.

### Control

All participants received UC, with those in the intervention arm receiving YPF in addition to UC. As there is no standardized treatment for this patient group, details of the type and frequency of UC received were collected via health economic data collection tools, primary care note reviews, and patient interviews.

### Procedure

Following baseline assessments, participants were randomized to the intervention or control group in block sizes of 4, to ensure similar numbers in each group, using an automated Web-based service provided by Bristol Randomised Trials Collaboration (independent clinical trials unit). The intervention group received an email with instructions on how to log-on using a unique username and password. Additional guidelines for YP and parents on how to make the most of YPF and support their child and a log to record health resource usage were emailed and posted. Participants were advised to complete all 7 weekly sessions consecutively but could choose to complete a session over 2 days. They were prompted to select a time for their next session via an embedded diary and sent automated reminders (to a parent/carer if preferred) via text and/or email 24 and 2 hours before their session was due. Automated text/emails reminded participants to complete homework if not completed 5 days after a session and invited participants to complete the *booster* quiz 6 weeks after session 7. At the end of sessions, participants could complete an embedded 2-min survey about their views of the session.

Control participants received an email or telephone call informing them of the allocation and emphasizing the importance of continued participation. During the trial, 4 newsletters were sent to all YP and parents to encourage engagement.

At 13, 26, and 52 weeks, YP and parents were emailed a link to a Web-based questionnaire hosted by www.qualtrics.com designed to take 30 min to complete. Noncompleters were prompted via email to complete questionnaires up to 3 times. After 13 (5 parents and 11 YP) or 52 weeks (3 parents, 5 YP, and 8 practice staff), participants were invited to share their experiences via a 30-min semistructured telephone interview.

### Outcomes

To inform future recruitment into a trial and YPF’s acceptability and safety, the study focused on comparison of recruitment rates via targeted letters, mass mail out, charities, and consultations; reasons YP with an appearance-altering condition declined participation; questionnaire completion rates and missing data; YPF acceptability (indicated by logged user statistics, session feedback, and percentage of YP/practice staff reporting login issues); YP and parent/carer views on YPF/UC; and the number and nature of safeguarding concerns and any action required. To determine the acceptability of the trial protocol, participants were asked about recruitment processes, random allocation, communicating with researchers, and safeguarding procedures. Proposed psychosocial outcome measures for the future definitive RCT were assessed at baseline and at 13, 26, and 52 weeks via online self-report questionnaires. Candidates for a primary outcome measure in the definitive trial were as follows:

1. 10-item Appearance Subscale from the Body Esteem Scale (BES-A) using a Likert scale (0=never to 4=always). Higher scores indicate greater appearance satisfaction. Scale reliability and validity have been previously demonstrated in adolescents [25]. In this study, the BES-A also showed strong internal consistency (alpha=.88).
2. 22-item Social Anxiety Scale (SAS) for adolescents using a Likert scale (1=not at all to 5=all the time). We used total SAS score and subscales scores for FNE, Social Avoidance and Distress in new situations (SAD-N) and in general situations, for example, with peers (SAD-G). Higher scores indicate greater anxiety. Scale reliability and validity have been previously demonstrated in adolescents [26]. In this study, the total SAS (alpha=.93), the FNE (alpha=.91), and the SAD-N (alpha=.86) also showed strong internal consistency. However, the internal consistency of SAD-G was comparatively less acceptable (alpha=.60).

Secondary outcome measures explored for their acceptability and sensitivity to change were as follows:

1. 5-item Romantic Appeal (RA) and 5-item Global Self-Esteem (SE) subscales from the Self-Perception Profile. YP choose which of 2 statements are “really true for me” =1 or “sort of true for me” =2 and decide whether the selected statement is “really true for me” =3 or “sort of true for me” =4. Higher scores indicate greater satisfaction with RA or higher SE. Scale reliability and validity have been previously demonstrated in adolescents [27]. In this study, the RA showed reasonable internal consistency (alpha=.68) and the SE good internal consistency (alpha=.77).
2. 21-item Perceived Stigmatization Questionnaire (PSQ) using a Likert scale with reversed scored items (never=5 or 1 to always=5 or 1). We calculated total PSQ score and subscales scores for absence of friendly behavior (AFB), confused and staring behavior (CSB), and hostile behavior (HB) by others. Higher scores indicate greater perceived stigmatization. Scale reliability and validity have been previously demonstrated in adolescents [28]. In this study, the total PSQ (alpha=.92), the CSB (alpha=.90), and the HB (alpha=.93) also showed strong internal consistency. However, the internal consistency of AFB was comparatively less acceptable (alpha=.68).
3. Communication, Cooperation, Assertion, Responsibility, Empathy, Engagement, and Self-control subscales (46 items) from the Social Skills Improvement System (SSIS) with a Likert scale (0=never to 3=almost always). Higher scores indicate greater perceived competence. Scale reliability and validity have been previously demonstrated in adolescents [29]. In this study, internal consistency scores for these subscales were good and ranged from alpha=.70 to alpha=.84.
4. Health-related quality of life was measured by the 5-level EuroQol-5D (EQ-5D-5L) questionnaire, a standardized instrument to measure generic health status for clinical and economic appraisal. The EQ-5D-5L has been validated in a diverse patient population in multiple countries [30]. Responses to this questionnaire are given utility values to produce a utility score for the health state quality-adjusted life years (QALY), which can be adjusted by weighting time spent in that health state by its utility score.

YP were asked if they had engaged in deliberate self-injury (DSI) over the past 3 months (no, once or twice, and 3 times or more). YPF was not designed to target DSI, but our previous evidence, suggesting DSI may be associated with appearance-related anxiety, demanded an assessment of its prevalence to determine if YPF should address this issue in the future. To establish the feasibility of collecting parent data as proxy indicators of their child’s well-being and the impact of the intervention, parents/carers completed parent versions of the SAS and SSIS at the same assessment points. YP were given a £10 Amazon voucher on completion of measures at 13, 26, and 52 weeks.

### Identifying and Measuring Resource Use

Resource use data were collected at 13, 26, and 52 weeks. Parents/carers completed an online study-specific Resource Use Questionnaire (RUQ) to collect data regarding all-cause and appearance-related health care and other resource use. The RUQ included questions on community-based contacts, including contacts with the GP, mental health nurse, psychologist, 111 service (UK telephone service for accessing nonemergency health care), school nurse, orthodontist, and mental health services; secondary care contacts with emergency, outpatient, and inpatient visits; contacts with social worker; charities; and personal costs accessing private services, make-up, and wig specialists and equipment. YP were also asked about days off school, which would potentially expand the future economic evaluation to take a societal perspective on costs. Those aged 16 and 17 years completed the RUQ if a parent/carer was not recruited. For comparison, study-specific case report forms were mailed to participants’ GP practices to report on health care resource use.

### Sample Size Considerations

No formal power calculations are undertaken in feasibility studies; instead, a suitable number of participants are recruited to gain knowledge about factors such as attrition and recruitment in relation to feasibility outcomes [31]. We aimed to recruit 60 YP to allow acceptability and completion rates to be estimated with error margins of ±13%, and with 1:1 randomization, 30 YP allocated to YPF would have in excess of 80% power for detecting a 50% or lower completion rate against an anticipated rate of 75%.

### Analysis

#### Acceptability of Intervention and Study Design

Descriptive statistics report YP sample characteristics; website use; and rates of recruitment, retention, and data completion. To inform acceptability of the chosen outcome measures, percentage missing values were determined at each assessment point, and qualitative feedback was collated from parents and YP via interviews. Interviews were digitally recorded and transcribed verbatim. Practice staff, parent, and YP data were analyzed separately using inductive thematic analysis [32]. Coding and theme development were driven by data content rather than existing concepts and involved: reading and becoming familiar with the full dataset; preliminary data coding to identify initial themes, which were clustered with a descriptive summary provided for each; and discussion of findings to reach consensus. Practice staff findings are published elsewhere [33], and only data relevant to the study objectives are reported here.

#### Health Economic Data Analysis

We applied the Devlin et al’s [34] UK preference weights for the 5L version to derive utility scores for YP, with the caveat these preference weights were developed for adults. We derived a 1-year QALY using the area under the curve method [35] and report QALY gain from baseline per trial arm. We derived rates of RUQ completion at 13, 26, and 52 weeks, compared resource use reported by participants and GP practices, and costed resources using of UK health and social care estimates of unit costs [36,37]. Analyses were performed in STATA v14.

#### Primary Outcome and Intervention Impact

The trial was not powered to test statistically significant impact; however, to inform the selection of a primary outcome measure and test for harm and potential effectiveness of YPF, the impact on repeated outcome measures was analyzed descriptively with some inferential methods used to describe the sample and estimate parameters. Statistical comparisons of outcomes were made between the 2 arms at 13-, 26-, and 52-week follow-up. Independent samples *t* test assessed if they differed at any given stage. Prior reasoning would suggest no or minimal systematic change in the control group and a high degree of correlation between baseline and follow-up data. If there is a systematic effect in the intervention group, there is the possibility that those at the worrying end of a scale may show greater change compared with those with relatively less worrying scores. Consequently, the rate of change in outcomes with baseline may differ between the 2 arms**.** Using analysis of covariance (ANCOVA), the groups were therefore compared on the primary outcome candidate measures allowing for initial commensurate baseline value (ie, main effect was *randomized group*, *baseline* was the covariate, and the interaction effect was group by covariate). For the intervention group, multiple regression considered outcome with respect to engagement (number of YPF sessions completed) after factoring in baseline position. At each stage, all available data were analyzed, and *P* values and partial eta-squared, a measure of effect size, are used to describe the data rather than confirm effects. Analyses were run using SPSS V23 (IBM).

## Results

### Recruitment Rate and Participants

**Figure 2:**
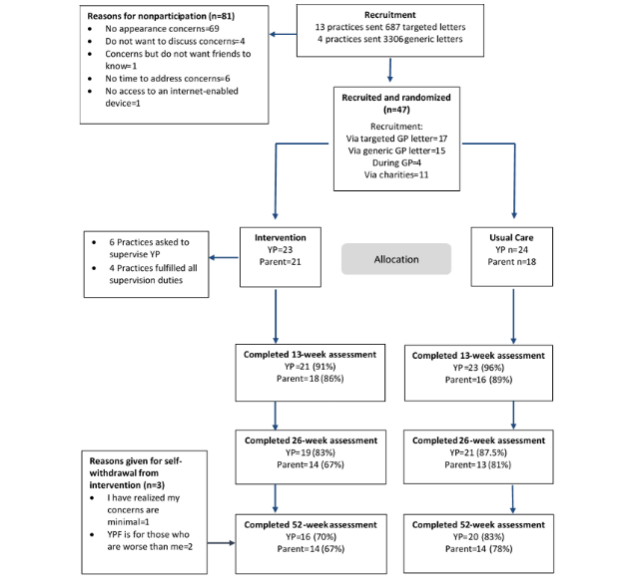
Consolidated Standards of Reporting Trials flow diagram. GP: general practitioner; YP: young people; YPF: Young Persons’ Face IT.

A total of 13 charities advertised the study once, resulting in 11 participants. A total of 13 practices in South West UK (practice sizes ranged from 3618-15,750 patients; mean 11,523, SD 3597), with a range of index of multiple deprivation (IMD) scores (1-10, where 1=10% most deprived), posted personalized invitations to 687 YP with an appearance-affecting condition. Identifying potential participants took 2 to 3 hours per practice. Overall, 17 YP consented to participate, giving a recruitment rate of 2.5%. Over 3 months, 4 additional GP practices (practice size=8314-10,726 patients; mean 9450, SD 8830) mass-mailed 3306 letters to all 12- to 17-year old patients, this took approximately 45 min per practice, and 15 YP consented to participate, giving a recruitment rate of 0.5% (Figure 2). Including this extension, recruitment was done from March to October 2015, and the last participant completed follow-up in September 2016.

YP and parents reported that letters from GPs provided credibility, with some expressing a preference for generic letters because YP were not singled out based on their difference and could decide if they had appearance-related distress. Practice staff preferred mass mail out over targeted letters because it was time efficient, and they found it difficult to judge patient suitability for targeted letters. In-consultation recruitment was low (n=4). Some staff found raising the option of appearance-related psychosocial support during consultations was difficult, especially when they perceived YP were expecting medical treatment only.

Overall, 47 Y*P* (26% male, 91% white; mean age 14.2 years, SD 1.7) from a range of socioeconomic backgrounds (IMD sample scores ranged from 1 to 10 with a mean of 6.78, SD 2.71) and with various conditions were randomized to YPF (n=23) or UC (n=24). In addition, 40 parents/carers were recruited. Demographic information is given in Table 2 and descriptive statistics for YP at all time points is given in Multimedia Appendix 1. Of 47 YP, at baseline, 25 (53%) reported being bullied. In comparison with population norms [25,26], 25 (53%) YP reported lower than average body esteem (mean 2.3, SD 0.8), 25 (53%) YP reported higher than average social anxiety (mean 44.5, SD 13.5), and 8 (17%, majority female) disclosed DSI.

**Table 2:** Key characteristics of young people at baseline.

Characteristics		Control (n=24)	Young Persons’ Face IT (n=23)
Age, mean (SD)		14 (1.95)	14 (1.42)
Female, n (%)		15 (63)	20 (87)
**Ethnicity, n (%)**			
	White British	20 (83)	23 (100)
	White other	—^a^	—
	Chinese	1 (4)	—
	Black African	—	—
	Black Caribbean	—	—
	Black British	—	—
	Indian	—	—
	Asian British	—	—
	Dual heritage	2 (8)	—
	Other	1 (4)	—
**Condition, n (%)**			
	Skin (eg, psoriasis and eczema)	11 (46)	11 (48)
	Craniofacial (eg, cleft and facial palsy)	5 (21	5 (22)
	Scarring (eg, burns and surgery)	3 (13)	4 (17)
	Birthmark (eg, port wine stain)	1 (4)	—
	Body form (eg, visible pacemaker, leg longer, missing finger, and fused toes)	4 (16)	3 (13)
**Deliberate self-injury^b^, n (%)**			
	Once or twice	1 (4)	4 (17)
	Thrice or more	1 (4)	2 (9)
	Total incidence (% female)	2 (100)	6 (60)

^a^Not applicable.

^b^Deliberate self-injury in the past 3 months.

### Reasons for Participation and Nonparticipation

Parents and YP cited lack of alternative support as a reason for participating:

I was hoping something like this would come our way one day.

parent, child with craniofacial condition

You can’t get help about these concerns.

female, 17 years, scars

The students that bullied me got offered counselling and I didn’t get anything!

female, 16 years, craniofacial condition

Of the 687 YP approached via targeted letters, 81 (11%) provided reasons for declining. Of these, 69 (85%) had no appearance concerns, 4 (5%) had concerns they did not wish to discuss, 6 (7%) had no available time, 1 (1%) did not want their friends to know, and 1 (1%) had no internet-enabled device.

### Acceptability of Study Design

Interviewees typically endorsed an RCT design:

I got UC, I didn’t really mind, as long as I was using my time to help.

female, 16 years, craniofacial condition

However, parents who cited lack of alternative support as a reason for participation reported their children were disappointed when allocated UC:

She really wanted to be the one that tried YP Face IT, so that was very disappointing.

parent, child with skin condition

Study newsletters and the facility to complete measures online were credited for maintaining study engagement:

The newsletters were really nice ... It keeps people engaged on my side of the study.

female, 17 years, Eczema, UC group

Questions were easy, I did them on my phone which was useful.

male, 12 years, skin condition

### Retention of Participants

In the intervention group, 3 patients self-withdrew. Of 3 patients, 1 decided the following after viewing YPF:

Helped me realise there are bigger problems and I could be a lot worse off, I’m happy the way I am.

female, 16 years, skin condition

Two felt it was more suitable for those with greater concerns:

It’s more for people that are very insecure and need help.

female, 15 years, birthmark

### Acceptability of Intervention and Safeguarding Processes

Table 3 details YPF usage and session feedback. The number of those attempting each session decreased as participants progressed through the intervention. Notably, of 23 patients, 12 (52%) attempted 7 sessions, and 9 (39%) completed the booster quiz. The time spent on each session by those who attempted it varied, from 1 (signed in to and left session) to 100 min, with a mean time ranging from 26.17 min (for session 7, which provides revision) to 47.60 min (session 2, which has the most content). Some completed a single session in 2 sittings. Percentage of session content viewed (an indication of adherence), by those attempting sessions, also varied and ranged from 10% to 100%. Sessions with the lowest completion rates were 1 (mean 87.13%) and 2 (mean 88.85%), but most of those who persisted with the program viewed all of the 7 sessions’ material (indicated by a median of 100%).

The only login errors and glitches reported (n=8) were with the booster quiz; these were addressed but accounted for 5 participants not completing the quiz. Of those attempting sessions, the majority agreed sessions were interesting, easy to understand and helpful. This was expanded on during interview:

It was really good, I found it very interesting listening to different ways of dealing with situations and the emotional side and sometimes you feel like you are the only one, but with YPF you know it’s not just you.

female, 14 years, scarring

Greatest variation in opinion was found in response to sessions 3 and 4 (managing challenging social interactions and challenging negative thoughts) where some indicated benefit from CBT more than SST and vice versa:

I had social skills... but YPF made me think, notice things which were positive, made me aware of things, like the subconscious, it’s a reminder that you’re not the centre of the world. People will look and go “ooh,” but then carry on. It made me not wait till it’s [skin condition] better and get on with life now.

male, 15 years

The bit on anxiety was really helpful.

male, 12 years, craniofacial condition

Some YP reported benefits from both:

The SCARED acronym was helpful and Testing the Water was good for starting small changes, like talking to people.

female, 14 years, craniofacial condition

YP reported that YPF validated their concerns and increased their confidence in seeking psychological support via primary care:

It’s made me aware that you can get help, I’d be more open to see a GP, and more comfortable talking about it now.

male, 13 years, skin condition

There were also suggestions that YPF affected decisions around appearance-altering surgery:

He’s been asking us to look into an aesthetic operation. We had the appointment after he had started YPF but he’s changed his mind and decided he doesn’t want it now, so YPF has been very useful.

parent, child with scars

Practice staff found supervision responsibilities brief (2-5 min per participant, per session) and straightforward, but only 59% of supervision tasks were completed, and forgetting and lack of time were barriers to completion. YP did not disclose safeguarding issues via YPF data collection tools, nor did they use the discussion forum. There was no evidence (from following up those who withdrew and analyses of outcome measures) of any intervention-related adverse events, but incidences of DSI at baseline were reviewed by the team’s clinical psychologist who adhered to NHS guidelines for its management. This resulted in 6 YP with DSI being advised to seek GP support, and in 2 cases, their GP was also informed via a letter.

The number of completed resource use categories over 1 year is small. Participants who completed questionnaires did not use some community-based services, such as GP nurse telephone calls and visits. Potential cost drivers of the intervention include GP visits, community mental health services, and secondary care visits. When asked about appearance-related resource use only, differences between arms were smaller, and fewer participants reported use. Resource use completion rates were higher using GP practices medical records review proformas. Practice staff completed these resources for 27 to 30 of the 47 patients in the trial, whereas only 19 patients self-reported these contacts.

**Table 3:** Young Persons’ Face IT intervention content and usage by participants (n=23) in the intervention group and online session feedback.

Session	Young people in intervention group attempting session, n (%)	Average minutes spent per session per person		Percentage of session content viewed per person		Median (minimum to maximum) response (1=strongly agree, 2=agree, 3=do not know, 4=disagree, and 5=strongly disagree)		
		Mean (SD)	Median (minimum to maximum)	Mean (SD)	Median (minimum to maximum)	Whether session was interesting	Easy to understand	Helped me
1	23 (100)	33.04 (26.80)	27 (1-100)	87.13 (24)	100 (28-100)	2 (1-2)	2 (2-2)	2 (1-2)
2	20 (87)	47.60 (26.10)	46 (6-90)	88.85 (24.82)	100 (10-100)	2 (1-3)	2 (1-2)	2 (1-2)
3	17 (74)	29.18 (21.76)	25 (2-76)	94.53 (14.78)	100 (42-100)	2 (1-3)	2 (1-2)	2 (1-3)
4	14 (61)	38.64 (23.69)	34.50 (14-83)	100 (0)	100 (100-100)	2 (1-3)	2 (1-3)	2 (1-3)
5	13 (57)	42.92 (25.25)	33 (13-91)	96.15 (7.68)	100 (80-100)	1 (1-1)	2 (2-2)	2 (1-2)
6	12 (52)	40.25 (23.95)	34 (6-89)	95.42 (8.91)	100 (75-100)	1 (1-2)	2 (2-2)	2 (1-3)
7	12 (52)	26.17 (18.64)	22.50 (5-67)	100 (0)	100 (100-100)	2 (1-2)	2 (1-2)	2 (1-2)
Quiz	9 (39)	31.33 (13.63)	30 (12-63)	100 (0)	100 (100-100)	—^a^	—	—

^a^Not applicable.

### Completion of Outcome and Resource Use Measures for Future Economic Evaluation

The percentage of participants providing data via online questionnaires at each assessment point was high for YP in both arms ranging from 96% to 70% with (76%) overall completion at 52 weeks, but there was a 13% comparative reduction in completion at 52 weeks among the intervention group (see Figure 1). Data completion was 100% for psychosocial measures. For the EQ-5D-5L, 70% (16/23) of patients in the YPF and 75% (18/24) in the UC group provided enough data to derive QALY. Completion of the online RUQ was more than 50% at 52 weeks for all categories, except community mental health services and days off school (Table 4). The control group provided more complete data than in the YPF group. Table 5 reports resource use for all medical reasons.

**Table 4:** Completeness of the 5-level EuroQol-5D and resource use data.

Number of completers of 5-level EuroQol-5D and resource use data	Young Persons’ Face IT (n=23), n (%)			Usual care (n=24), n (%)		
	Week 13	Week 26	Week 52	Week 13	Week 26	Week 52
5-level EuroQol-5D	21 (91)	19 (83)	16 (70)	23 (96)	21 (88)	20 (83)
Quality-adjusted life years complete cases	—^a^	—	16 (70)	—	—	18 (75)
General practitioner services	13 (57)	13 (57)	15 (65)	19 (79)	14 (58)	18 (75)
Mental health services	7 (30)	2 (9)	9 (39)	8 (33)	7 (29)	13 (54)
Social services	13 (57)	13 (57)	15 (65)	19 (79)	14 (58)	18 (75)
Other National Health Services Community services	13 (57)	13 (57)	15 (65)	19 (79)	14 (58)	18 (75)
Outpatient appointments	17 (74)	16 (70)	12 (52)	21 (88)	16 (67)	13 (54)
Accident and emergency	19 (83)	16 (70)	14 (61)	21 (88)	17 (71)	16 (67)
Inpatient nights	19 (83)	16 (70)	16 (70)	21 (88)	17 (71)	17 (71)
Hospital tests	19 (83)	16 (70)	16 (70)	21 (88)	16 (67)	18 (75)
Private services/expenses	19 (83)	15 (65)	15 (65)	19 (79)	16 (67)	17 (71)
Days off school	7 (30)	9 (39)	8 (35)	10 (42)	10 (42)	6 (25)
Resource complete cases	5 (22)	2 (9)	5 (22)	5 (21)	5 (21)	9 (38)

^a^Not applicable.

**Table 5:** Number of participants who completed the resource use questions at each time points, the number who used the resource, the mean units of resource used, and their mean costs.

Resource	Young Persons’ Face IT				Usual care			
	N^a^	N^b^>0	Resource use, mean (SD)	Cost (£), mean, (SD) (£)	N^a^	N^b^>0	Resource use, mean (SD)	Cost (£), mean (SD)
GP^c^ visits	6	3	3.0 (3.3)	132 (147)	13	9	2.0 (2.3)	88 (103)
GP calls	6	1	0.2 (0.4)	5 (11)	13	2	0.7 (2.2)	19 (60)
GP home visits	6	0	0.0	0.0	13	0	0.0	0.0
GP nurse visits	6	2	1.3 (2.2)	19 (31)	13	4	0.7 (1.2)	10 (17)
GP nurse calls	6	0	0.0	0.0	13	0	0.0	0.0
GP nurse home visits	6	0	0.0	0.0	13	0	0.0	0.0
Mental health nurse	6	0	0.0	0.0	13	1	0.2 (0.6)	7 (24)
Psychologist	6	0	0.0	0.0	13	3	0.6 (1.3)	86 (184)
111 calls	6	0	0.0	0.0	13	0	0.0	0.0
School nurse	6	2	0.5 (0.8)	6 (11)	13	2	0.2 (0.6)	3 (8)
Orthodontist	6	3	1.7 (1.9)	167 (186)	13	5	0.6 (1.1)	62 (112)
Mental health services	0	0	0.0	0.0	6	1	0.7 (1.2)	75 (185)
Outpatient appointments	11	5	N/A^d^	199 (312)	14	5	N/A	210 (486)
Accident and emergency visits	13	3	0.3 (0.6)	41 (83)	16	4	0.3 (0.6)	41 (79)
Inpatient nights	13	0	0.0 (0.0)	0 (0)	16	1	0.1 (0.3)	22 (89)
Social worker contacts	6	0	0.0	0.0	13	0	0.0	0.0
Charity contacts	6	0	0.0	0.0	13	1	0.2 (0.6)	0 (0)
Private counseling	13	2	1.8 (6.4)	58 (191)	15	1	0.2 (0.8)	N/R^e^
Private services	13	2	0.3 (0.9)	5 (19)	15	0	0.0	0.0
Make-up and wig specialist	13	0	0.0	0.0	16	0	0.0	0.0
Make-up, wigs, and other equipment	12	1	0.0	1 (3)	13	1	0.1 (0.3)	4 (13)

^a^Number of people who completed the resource use question at 13, 26, and 52 weeks allowing for a 1-year cost to be derived.

^b^Of those who completed, number of participants who reported having used the resource.

^c^GP: general practitioner.

^d^Not applicable. A mix of different appointments at different costs reported.

^e^Not reported, missing data.

### Variation of Usual Care

Participants were asked to record any psychosocial support they received for appearance concerns. One reported receiving support from a private counselor and one from an NHS counselor, both were in the UC arm.

### Selecting Primary Outcome Measure and Estimate of Impact on Outcome Measures

Independent samples *t* tests at 13, 26, and 52 weeks did not show statistically significant differences between the 2 arms on any measure. Positive changes to the primary outcome candidate measures in the intervention arm (BES-A and the FNE subscale of the SAS) were found when factoring in baseline scores and engagement with the program (see Tables 6 and 7).

After adjusting for BES-A baseline scores, there were statistically significant main effects for randomized group at 13 weeks (*P*=.001), 26 weeks (*P*=.001), and 52 weeks (*P*=.02) and interaction effects at 13 weeks (*P*<.001), 26 weeks (*P*=.002), and 52 weeks (*P*=.006). Engagement with the intervention was a significant predictor of BES-A scores at 13 weeks (*P*=.02) and 26 weeks (*P*<.001), but this was not maintained at 52 weeks (*P*=.29). After adjusting for FNE baseline scores, there were statistically significant main effects for randomized group at 13 weeks (*P*=.05) and 26 weeks (*P*=.02) and interaction effects at 13 weeks (*P*=.03) and 26 weeks (*P*=.007), but no statistically significant main (*P*=.29) or interaction (*P*=.22) effects at 52 weeks. Engagement with the intervention was a significant predictor of FNE scores at 13 weeks (*P*=.01) and 26 weeks (*P*=.01), but again this was not maintained at 52 weeks (*P*=.25).

Although the study was not powered to confirm effects, results suggest that YPF may improve BES-A and FNE for those at the worrying end of these scales, and that increased engagement with YPF may be a contributory factor.

The BES-A would be an appropriate primary outcome measure for a future RCT. The BES-A is frequently used in adolescent body image research because it is reliable, has normative data, and has good face validity among adolescents (eg, a study by Diedrichs et al [38]); it provides a general measure of satisfaction with appearance and is not condition specific, making it appropriate for those with any appearance-affecting condition. In this study, YP fed back that it was quick and easy to complete, and results indicated it is sensitive to change among those completing the intervention.

The Consolidated Standards of Reporting Trials-electronic health checklist is provided in Multimedia Appendix 2.

**Table 6:** Change in appearance and social anxiety outcomes at each time point and between each arm when factoring in baseline values.

Assessment point, measure		Valid (n)	Main effect for randomized group		Measure at baseline		Interaction	
			*P* value	η_p_^2a^	*P* value	η_p_^2^	*P* value	η_p_^2^
**13 weeks**								
	BES-A^b^	44	.001	0.253	<.001	0.585	<.001	0.287
	SAD-N^c^	44	.08	0.071	<.001	0.534	.09	0.068
	FNE^d^	44	.04	0.095	<.001	0.593	.03	0.108
	SAD-G^e^	44	.91	0	<.001	0.453	.91	0
**26 weeks**								
	BES-A	40	.001	0.257	<.001	0.388	.002	0.242
	SAD-N	40	.005	0.203	<.001	0.422	.001	0.255
	FNE	40	.02	0.135	<.001	0.29	.007	0.187
	SAD-G	40	.23	0.039	.002	0.229	.05	0.099
**52 weeks**								
	BES-A	36	.02	0.153	<.001	0.445	.006	0.212
	SAD-N	36	.14	0.065	<.001	0.526	.08	0.088
	FNE	36	.29	0.034	.002	0.273	.22	0.046
	SAD-G	36	.57	0.01	<.001	0.356	.27	0.037

^a^Thresholds for partial eta-squared η_p_^2^: <0.0025 indicates a trivial inconsequential effect, 0.0025 to 0.01 indicates a small effect, 0.01 to 0.06 indicates a moderate effect, 0.06 to 0.14 indicates a medium-sized effect, 0.14 to 0.30 indicates a large effect, 0.30 to 0.50 a very large effect, and >0.50 indicates a huge effect.

^b^BES-A: Body Esteem Appearance subscale.

^c^SAD-N: Social Avoidance and Distress in New situations.

^d^FNE: Fear of Negative Evaluation.

^e^SAD-G: Social Avoidance and Distress among peers.

**Table 7:** The impact of engagement with the Young Persons’ Face IT intervention on appearance and social anxiety outcomes at each time point when factoring in baseline value.

Assessment point, measure		Valid (n)	R^2^^a^	Baseline measure		Engagement	
				Beta	*P* value	Beta	*P* value
**13 weeks**							
	BES-A^b^	21	0.396	.427	.03	.461	.02
	SAD-N^c^	21	0.340	.627	.007	−.158	.45
	FNE^d^	21	0.574	.637	.001	−.420	.01
	SAD-G^e^	21	0.439	.677	.001	−.173	.35
**26 weeks**							
	BES-A	19	0.682	.057	.69	.816	<.001
	SAD-N	19	0.371	.430	.05	−.581	.01
	FNE	19	0.337	.070	.73	−.571	.01
	SAD-G	19	0.349	.217	.29	−.557	.01
**52 weeks**							
	BES-A	16	0.202	.282	.27	.323	.21
	SAD-N	16	0.438	.684	.008	−.331	.15
	FNE	16	0.216	.344	.18	−.295	.25
	SAD-G	16	0.285	.561	.04	−.292	.26

^a^R*2*: indicates the proportion of variation in outcome jointly accounted for by the baseline measure and level of engagement.

^b^BES-A: Body Esteem Appearance subscale.

^c^SAD-N: Social Avoidance and Distress New situations.

^d^FNE: Fear of Negative Evaluation.

^e^SAD-G: Social Avoidance and Distress among peers.

### Recruitment for Full Randomized Controlled Trial

A future RCT design would be amenable to analysis using ANCOVA with a baseline by group interaction, and 53, 70, and 86 participants per arm would have 80%, 90%, and 95%, respectively, power for detecting anticipated effects; this power is supported by lower bounds on effect sizes from this feasibility study. This study indicates 76% full data completion at 52 weeks, recruiting 186 participants will give complete data on 140 participants (90% power).

## Discussion

### Principal Findings

This study explored the feasibility of using an RCT to evaluate the effectiveness and cost-effectiveness of YPF, an online psychosocial intervention to support YP with appearance-related anxiety. Results indicate YPF is a welcome, safe, and acceptable intervention with the potential to fill a gap in care provision and suggest an RCT design would be acceptable and feasible with wide-ranging recruitment strategies, using the BES-A subscale as primary outcome measure.

Lessons learned will inform a future RCT, particularly around engaging YP in appearance-related research, an extremely sensitive topic rarely discussed with adults in primary care settings [18]. Recruiting from this group is notoriously challenging [39], and pertinent barriers and facilitators to recruitment identified in this study are discussed in detail elsewhere [33]. In summary, educating staff on the importance of normalizing conversations about appearance and validating rather than minimizing concerns in primary care settings could increase YP help-seeking behavior and reduce perceived stigma around receiving psychosocial support. Despite these challenges, recruitment via charitable organizations and GP practices is feasible; but to achieve the recommended large trial sample size, in addition to advertising via a wide range of relevant charities, using social media and a mass mail out approach from large GP practices is recommended. This would also allow YP to decide whether or not their condition causes psychological distress, rather than GPs judging their suitability; which in this study often involved GPs second-guessing the objective severity of the visible difference. This recommendation aligns with evidence that an individual’s subjective assessment of the impact of a visible difference is a better predictor of adjustment [14] and recommendations that health professionals should ask about, rather than assume, levels of distress [40].

The majority of YP found YPF sessions interesting and helpful, and retention and data completion strategies (eg, online questionnaires and text reminders) were largely successful. Retention (76% of all YP completed data at 52 weeks) and intervention-adherence rates (52% completed the program) were comparable with that demonstrated in similar studies using internet-based CBT for adolescent anxiety [41]. Nonetheless, and particularly considering indications that increased engagement may improve outcomes, adherence could be improved. Feedback that YPF may not suit all (eg, some felt it was suited to those with greater concern) suggests that more stringent inclusion criteria based on level of distress could be employed in future. However, given evidence that YPF does not cause harm, the preliminary nature of these findings and our aim to provide easily accessible support for all who want it, at this stage, we recommend retaining current inclusion criteria and incorporating a subset analysis for those who score highly at baseline.

Although the potential benefits and nature of blended care (a combination of electronic health and guidance from a care provider) are being debated [42], definitive trials could also consider preventing attrition by including, for example, a telephone call from the supervising health professional to YP who do not progress as expected or support from a peer who has completed the program. Qualitative data suggest that depending on individual needs, some YP may benefit from additional motivation and support. However, the YPF forum, an opportunity to gain peer support and included on request from our YP advisory group, was not used. The value of this feature should be confirmed in a larger trial.

The safeguarding protocol for ensuring vulnerable YP were followed up by the research team was successful. Whether it is feasible or necessary for practice staff to review YP data weekly is undecided; insufficient time/forgetting resulted in some staff failing to review accounts. However, as it appears that YP do not disclose safeguarding issues via the website (all cases of DSI were reported in response to a single item within outcome measures), it may be more feasible for researchers to continue with weekly checks (to confirm this finding) while determining whether automated reminders to staff to review patient data increases adherence. These data could ultimately provide GPs with information to determine the need for a follow-up appointment after the YP has completed YPF. Finally, to replace a task fulfilled by the team’s clinical psychologist in this study, in future trials, YP will be signposted to appropriate sources of support for DSI within YPF.

We found that resource use data collection via online questionnaires is potentially burdensome, and completion rates are low. Patients reported the use of resources beyond the health and social care payer perspective, with high costs of private counseling and other expenses. A future economic evaluation could include a private perspective on costs and should rely on resources being completed through GP practice proformas, complemented by participant self-report on the use of private and other mental health services. Findings from the qualitative study also highlight that the follow-up of the future RCT will need to be long enough to capture potential long-term health care savings accruing from YPF, such as cosmetic surgeries and other expensive treatments avoided.

### Strengths

YPF is an innovative, easily accessible intervention with the potential to improve outcomes for YP with a visible difference and appearance-related distress who currently have limited access to evidence-based specialist support. Extensive reflection and user involvement built into the study design, identified a feasible recruitment strategy that ultimately provided sufficient data to address study objectives and inform the design of future trials. Independent randomization and use of well-established outcome measures ensured data were reliable and valid, and a primary outcome measure (BES-A) was selected.

### Limitations

As there is no best alternative therapy available for YP with a visible difference, apart from limited access to a mental health practitioner, there was no active control arm. Although our initial concerns that YP randomized to receive UC may be disappointed were borne out, there was minimal evidence that this deterred participation. However, considering this disappointment and confirmation that there is little alternative support available, future trials should consider a wait-list control arm. A higher dropout in the YPF arm may have resulted from the increased burden associated with completing the intervention. Participants required an internet-enabled device, which may have restricted access to those with lower socioeconomic status; although only 1 person identified this as a reason for declining involvement, this issue requires consideration. The majority ethnicity of the sample was white, which reflects a typical bias across appearance research [43] that needs addressing in future studies. Finally, we relied on self-report measures that may result in reporting bias, and YP were not blinded to their allocation.

### Conclusions

We successfully delivered a novel online intervention for YP disclosing appearance-related distress associated with an appearance-altering condition and confirmed the feasibility of evaluating it against a UC control group using an RCT design, with high levels of data completeness and reasonable intervention adherence. Despite reporting a range of negative appearance-related experiences, including bullying, self-harm, poor body esteem, and social anxiety, participants had not sought appearance-related support or known how to do so. YPF may prove to be a feasible, cheap, and acceptable source of immediate specialist support, particularly for those with low body esteem and high levels of social anxiety. YP involved in the development of YPF coproduced a video summarizing this study, available on YouTube [44].

## Appendix

Multimedia Appendix 1 Descriptive statistics on young people’s outcome measures at all time points.

Multimedia Appendix 2 CONSORT-EHEALTH checklist (V 1.6.1).

## Abbreviations

ANCOVA: analysis of covariance
BES-A: Body Esteem Appearance subscale
BID: body image dissatisfaction
CBT: cognitive behavioral therapy
DSI: deliberate self-injury
EQ-5D-5L: 5-level EuroQol-5D
FNE: Fear of Negative Evaluation subscale
GP: general practitioner
HB: hostile behavior
IMD: Index of Multiple Deprivation
NHS: National Health Services
PSQ: Perceived Stigmatization Questionnaire
PTSD: posttraumatic stress disorder
QALY: quality-adjusted life years
RA: romantic appeal
RCT: randomized controlled trial
RUQ: Resource Use Questionnaire
SAD-G: Social Anxiety in General Situations subscale
SAD-N: Social Anxiety in New Situations subscale
SAS: Social Anxiety Scale
SE: Self-Esteem
SSIS: Social Skills Improvement System
SST: social skills training
UC: usual care
YP: young people
YPF: Young Persons’ Face IT
