# Supplementary material for: A Web-Based Self-Help Psychosocial Intervention for Adolescents Distressed by Appearance-Affecting Conditions and Injuries (Young Persons’ Face IT): Feasibility Study for a Parallel Randomized Controlled Trial
Source: JMIR Ment Health. 2019 Nov 22;6(11):e14776. doi: 10.2196/14776 (PMC6898888; doi:10.2196/14776)
Supplement: Multimedia Appendix 1 [file mental_v6i11e14776_app1.docx]

| Variable | Cronbach alpha | Group | Baseline (control, n=24; YPF^a^, n=23) | 13 weeks (control, n=23; YPF, n=21) | 26 weeks (control, n=21; YPF, n=19) | 52 weeks (control, n=20; YPF, n=16) |
| --- | --- | --- | --- | --- | --- | --- |
| Body Esteem Appearance, mean (SD) | .88 | Control | 2.45 (0.83) | 2.40 (0.96) | 2.24 (1.09) | 2.69 (0.90) |
|  | —^b^ | YPF | 2.07 (0.72) | 2.09 (0.54) | 2.19 (0.81) | 2.31 (0.55) |
| Social Anxiety Scale total, mean (SD) | .93 | Control | 42.47 (14.01) | 40.39 (14.46) | 41.86 (15.34) | 38.70 (14.61) |
|  | — | YPF | 45.71 (13.03) | 42.33 (10.51) | 38.58 (12.11) | 37.94 (11.74) |
| Fear of Negative Evaluation, mean (SD) | .91 | Control | 19.65 (7.64) | 18.48 (8.34) | 19.24 (8.32) | 17.10 (7.70) |
|  | — | YPF | 21.81 (7.06) | 20.0 (5.78) | 18.11 (7.12) | 17.25 (5.59) |
| Social Avoidance and Distress in New Situations, mean (SD) | .86 | Control | 15.78 (5.33) | 15.0 (5.25) | 14.86 (5.97) | 14.13 (4.70) |
|  | — | YPF | 16.19(5.60) | 15.71 (5.01) | 14.05 (3.69) | 14.12 (4.70) |
| Social Avoidance and Distress among peers, mean (SD) | .60 | Control | 7.04 (2.50) | 6.91 (2.59) | 7.76 (2.98) | 7.45 (2.96) |
|  | — | YPF | 7.86 (2.03) | 7.10 (2.28) | 6.42 (2.32) | 6.56 (2.37) |
| Romantic Appeal, mean (SD) | .68 | Control | 2.54 (0.66) | 2.47 (0.73) | 2.51 (0.81) | 2.95 (0.81) |
|  | — | YPF | 2.21 (0.66) | 2.31 (0.57) | 2.45 (0.48) | 2.56 (0.60) |
| Self-esteem, mean (SD) | .77 | Control | 2.98 (0.66) | 3.07 (0.78) | 2.90 (0.94) | 3.17 (0.74) |
|  | — | YPF | 2.63 (0.83) | 2.70 (0.76) | 2.75 (0.84) | 3.08 (0.79) |
| Perceived Stigmatization Questionnaire, mean (SD) | .92 | Control | 2.12 (0.57) | 2.01 (0.54) | 2.06 (0.59) | 1.92 (0.54) |
|  | — | YPF | 2.25 (0.61) | 2.13 (0.52) | 1.94 (0.59) | 1.96 (0.52) |
| Absence of Friendly Behavior, mean (SD) | .68 | Control | 2.30 (0.42) | 2.12 (0.56) | 2.39 (0.46) | 2.33 (0.47) |
|  | — | YPF | 2.35 (0.36) | 2.06 (0.37) | 2.21 (0.43) | 2.25 (0.47) |
| Confused and Staring Behavior, mean (SD) | .90 | Control | 2.04 (0.76) | 1.78 (0.65) | 1.78 (0.63) | 1.60 (0.61) |
|  | — | YPF | 2.06 (0.84) | 1.98 (0.83) | 1.72 (0.78) | 1.80 (0.78) |
| Hostile Behavior by Others, mean (SD) | .93 | Control | 1.90 (0.99) | 1.77 (0.90) | 1.97 (0.94) | 1.77 (0.99) |
|  | — | YPF | 2.29 (0.95) | 2.10 (0.73) | 1.85 (0.88) | 1.78 (0.67) |
| Communication, mean (SD) | .78 | Control | 14.75 (2.45) | 15.39 (2.41) | 14.67 (2.31) | 15.15 (2.62) |
|  | — | YPF | 13.78 (3.10) | 13.71 (2.41) | 14.05 (2.82) | 14.81 (2.83) |
| Cooperation, mean (SD) | .79 | Control | 15.12 (3.27) | 15.95 (3.45) | 15.09 (3.56) | 15.7 (4.50) |
|  | — | YPF | 13.95 (3.45) | 13.66 (3.16) | 14.21 (3.03) | 15.25 (2.79) |
| Assertion, mean (SD) | .73 | Control | 12.25 (3.52) | 13.69 (3.64) | 12.71 (3.69) | 13.15 (4.73) |
|  | — | YPF | 12.08 (4.18) | 13.04 (3.93) | 13.37 (4.04) | 14.00 (4.06) |
| Responsibility, mean (SD) | .70 | Control | 15.58 (2.50) | 15.95 (3.15) | 16.38 (2.67) | 16.3 (3.29) |
|  | — | YPF | 14.86 (3.42) | 15.38 (3.13) | 15.05 (4.03) | 15.87 (3.66) |
| Empathy, mean (SD) | .84 | Control | 14.79 (2.78) | 13.85 (2.85) | 14.19 (2.50) | 15.25 (2.75) |
|  | — | YPF | 14.00 (3.31) | 14.30 (2.81) | 14.31 (3.23) | 14.75 (2.59) |
| Engagement, mean (SD) | .75 | Control | 14.16 (2.89) | 15.21 (3.66) | 14.67 (3.95) | 15.55 (4.61) |
|  | — | YPF | 13.13 (4.24) | 13.90 (4.10) | 14.53 (4.06) | 14.56 (4.56) |
| Self-control, mean (SD) | .84 | Control | 10.62 (3.28) | 11.86 (10.85) | 11.43 (2.04) | 12.15 (3.88) |
|  | — | YPF | 10.56 (4.19) | 18.85 (3.33) | 12.53 (2.95) | 13.18 (3.98) |

^a^YPF: Young Persons’ Face IT.

^b^—: not applicable
